# Oral Abstracts of scientific contributions to GCOM 2019: 3rd Global Conference on Myositis. Berlin, Germany. 27-30 March 2019

**DOI:** 10.1186/s41927-020-00135-6

**Published:** 2020-07-06

## Oral Presentations

### **Oral1 Xenograft model of sporadic inclusion body myositis**

#### K. Britson

##### Johns Hopkins University, Baltimore, MD, United States

Objectives: Fundamental obstacles to the development of therapies for sporadic Inclusion Body Myositis (IBM) include our limited understanding of disease pathogenesis as well as a lack of animal models. The robust endomysial inflammation, an increased association of IBM with specific HLA haplotypes and other autoimmune diseases, and the presence of autoantibodies in many patients suggests that IBM is primarily an autoimmune disease. However, an association with aging, a lack of response to immunotherapy, and presence of ubiquitinated protein aggregates suggest the immune response may be secondary to myodegeneration. We hypothesize that our xenograft model will allow us to elucidate the contributions of inflammation and myodegeneration in the pathogenesis of IBM and may be a useful preclinical model for testing novel therapies.

Methods: In this xenograft model, human muscle biopsy specimens are transplanted into immunodeficient mice. The human myofibers cut during the biopsy procedure degenerate, but new muscle fibers regenerate from the patient’s satellite cells resulting in nascent human myofibers for study. This newly regenerated muscle is revascularized and innervated by the mouse host. Xenografts are collected at various post-operative timepoints ranging from one to eleven months and cryosectioned to carry out histochemical and immunohistochemical analysis.

Results: IBM xenografts develop both inflammatory and degenerative pathologic features of the human disease. At 4 months, collections of xenografts from a patient with a normal muscle biopsy, a dermatomyositis patient, and multiple IBM patients display successful regeneration. Regeneration appears less robust in IBM xenografts and is inversely associated with the number of human CD3+ T cells and sarcoplasmic MHC-I upregulation. Co-staining of CD8 and Ki-67 reveal that a majority of the CD8+ T cells within the IBM xenografts are proliferative at 4 months, and T cell receptor sequencing shows that T cells within the xenografts are oligoclonal. Preliminary experiments indicate that removal of these immune cells via low dose irradiation improves regeneration of IBM xenografts. In addition to these inflammatory features, at later timepoints, missplicing defects due to loss of nuclear TDP-43 can be detected, and IBM xenografts show rare fibers containing p62 positive aggregates.

Conclusions: This mouse xenograft model shows characteristic features of the human disease including protein aggregation and endomysial inflammation. We are using this model for mechanistic studies and preclinical therapeutic testing in IBM.

### **Oral2 Targeting highly differentiated effector memory KLRG1+ cytotoxic T cells in inclusion body myositis**

#### S. Greenberg

##### Brigham and Woman's Hospital, Boston, MA, United States

Inclusion body myositis (IBM) is a late onset progressive inflammatory disorder of skeletal muscle. Its autoimmune basis is indicated by its association with a blood autoantibody, anti-cN1A, genome-wide studies showing marked association (P=3.58x10-33) with an HLA autoimmune haplotype, and muscle biopsy pathology, characterized by widespread myofiber expression of MHC class 1 and 2 molecules, an intense cytokine environment, and the destruction of myofibers by cytotoxic CD8+ T cells. The refractory nature of IBM has led in part to speculation that it is not an autoimmune disease. An alternative explanation for its refractoriness may come from the inability of conventional immunosuppressive therapies to address the specific nature of the T cell autoimmunity present in IBM Here, we report on translational studies of IBM patient muscle (N=39) and blood samples (N=12) compared with large numbers (N=299) of samples from other muscle diseases, identifying a unique cytotoxic lymphocyte signature in IBM and highlighting the relevance of highly differentiated cytotoxic T cells to the pathogenesis of IBM. We further identified through a bioinformatics analysis a strategy of selective depletion of this population of cells through targeting the surface receptor killer cell lectin-like receptor G1 (KLRG1), and describe the preclinical development of potent therapeutic depleting anti-human KLRG1 antibodies and validate their selective depletion in non-human primate studies.

### **Oral3 CD8+T-bet+ cells as a predominant biomarker for inclusion body myositis**

#### G. Dzangué-Tchoupou

##### Centre of Research in Myology, Paris, France

Background: Myositis is a heterogeneous group of muscular auto-immune diseases with clinical and pathological criteria that allow the classification of patients into different sub-groups. Inclusion body myositis is the most frequent myositis above fifty years of age. Diagnosing inclusion body myositis requires expertise and is challenging. Little is known concerning the pathogenic mechanisms of this disease in which conventional suppressive-immune therapies are inefficacious.

Objectives: Our aim was to deepen our understanding of the immune mechanisms involved in inclusion body myositis and identify potential biomarkers.

Methods: Using a panel of thirty-seven markers and mass cytometry, we performed deep immune profiling of peripheral blood cells from inclusion body myositis patients and healthy donors, divided into two cohorts: test and validation cohorts. Potential biomarkers were compared to a pool of myositis control patients (anti-Jo1-, anti-3-hydroxyl-3-methylglutaryl CoA reductase-, and anti-signal recognition particle-positive patients). Results: Unsupervised analyses revealed substantial changes only within CD8+ cells. We observed an increase in the frequency of CD8+ cells that expressed high levels of T-bet, and containing mainly both effector and terminally differentiated memory cells. The senescent marker CD57 was overexpressed in CD8+T-bet+ cells of inclusion body myositis patients compared to healthy donors. As expected, senescent CD8+T-bet+ CD57+ cells of both patients and healthy donors showed a loss of co-stimulatory molecules, and had the immune profile CD28nullCD27nullCD127null. Surprisingly, non-senescent CD8+T-bet+ CD57- cells in inclusion body myositis patients expressed lower levels of CD28, CD27, and CD127, and expressed higher levels of activation markers such as CD38 and HLA-DR compared to healthy donors. Using classification and regression trees alongside receiver operating characteristics curves, we identified and validated a frequency of CD8+T-bet+ cells > 51.5% as a diagnostic biomarker specific to inclusion body myositis, compared to myositis control patients, with a sensitivity of 94.4%, a specificity of 88.5%, and an area under the curve of 0.97.

Conclusion: Using a panel of thirty-seven markers by mass cytometry, we identify a specific activated cell population (CD8+T-bet+ CD57- CD28lowCD27lowCD127low CD38+ HLA-DR+), which could play a role in the physiopathology of inclusion body myositis. We also identify a novel biomarker: CD8+T-bet+ cells, as a predominant biomarker of this disease in comparison to 3 other groups of myositis patients.

### **Oral4 Overall and site-specific cancer risk before and after diagnosis of idiopathic inflammatory myopathies: Novel register data from 2002 to 2016 in Sweden**

#### L. Dani

##### Karolinska Institute and Karolinska University Hospital, Stockholm, Sweden

Objectives: to update knowledge on the cancer risk in idiopathic inflammatory myopathies (IIM) compared to the general population before and after IIM diagnosis.

Methods: population-based matched case-control (before IIM diagnosis) and population-based matched cohort (after IIM diagnosis) study with data from the National Patient Register between 2002 and 2016, the Swedish Cancer Register and the Cause-of-Death Register. We analyzed the association between sex, age at diagnosis, IIM subtypes and overall or site-specific cancer risk in a defined time interval within a long follow-up period before and after IIM diagnosis, using logistic regression and Cox regression model.

Results: We identified 1419 patients (79 with JDM, 471 with DM, 869 with other IIM) with IIM diagnosis between 2002 and 2016 in Sweden and matched them to 7045 general references. Sixty percent were women, and more than two-thirds were diagnosed after 49 years of age. Before IIM diagnosis, there was a 51% increased occurrence of any first primary cancer in patients who later developed IIM compared to the general references. The increased occurrence was even more apparent within one year before and 90 days after IIM diagnosis (Adjusted Odds Ratio [AOR] 4.39, 95% Confidence Interval [CI] 3.18-6.06). Only cancers of digestive organs and peritoneum (AOR 2.06, 95% CI 1.36-3.14), respiratory system (AOR 4.19, 95% CI 2.10-8.35), skin (AOR 1.57, 95% CI 1.03-2.40), lymphatic and hematopoietic tissue (AOR 3.68, 95% CI 2.03-6.69) were significantly associated with IIM. The adjusted hazard ratio (AHR) of cancer comparing IIM patients with the reference population after diagnosis was 1.66 (95% CI 1.38-2.00). Being a man, having DM and age between 19 to 49 resulted in higher risk. After IIM diagnosis, the overrepresented cancer types were cancers of the buccal cavity and pharynx (AHR 9.81, 95% CI 3.46-27.85), female genital organs (AHR 2.23, 95% CI 1.21-4.09), skin (HR 2.99, 95% CI 2.05-4.34), lymphatic and hematopoietic tissue (HR 2.70, 95% CI 1.44-5.07). Higher cancer risk was observed within the three years before and after IIM diagnosis, but we also found significant cancer risk within three to ten years after IIM diagnosis.

Conclusion: Cancer risk in IIM patients increases before diagnosis and reaches its top one year before diagnosis and continues to be significant until 10 years later. Risk for site-specific cancer varies before and after IIM diagnosis being highest for respiratory and digestive organs before diagnosis and buccal cavity after. This has implications for our clinical management of these patients.

### **Oral5 Immunization of dermatomyositis-specific auto-antigen transcriptional intermediary factor (TIF1)-γ induces myositis in mice**

#### N. Okiyama

##### University of Tsukuba, Ibaraki, Japan

Objectives: While murine models of experimental myositis have been established using immunizations of muscle-specific proteins like myosin and C protein, they are not targeted in actual diseases. By contrast, a number of myositis-specific autoantigens recently have been identified, although their relevance to the pathogenesis remains unclear. Here we established a new murine model of experimental myositis inducible with immunizations of TIF1-γ, one of self-antigens for myositis-specific autoantibodies, and analyzed the pathogenesis.

Methods: We purified recombinant whole human TIF1-γ protein using baculovirus expression systems in insect cells, and immunized wild-type, 32microglobline-knockout, perforin-knockout, and tMT C57BL/6 (B6) mice with subcutaneous injections of emulsions containing the protein and complete Freund's adjuvant four times and with intraperitoneal injections of pertussis toxin. Myositis with broken muscle fibers was observed in bilateral muscles of the immunized mice 14 days after the last immunizations. For adaptive transfer experiments, the T cells from the immunized mice co-cultured ex vivo with lipopolysaccharide-activated bone marrow-derived dendritic cells presenting TIF1-γ, and then purified to CD8+ or CD4+ T cells using MACS® systems. IgG were collected form the sera of TIF1-γ-immunized mice using protein G columns.

Results: The lymph node T cells from the immunized mice proliferated when co-cultured with dendritic cells presenting TIF1-γ, and IgG reacting TIF1-γ were detected in the sera of the immunized mice. Transfer of the T cells, which proliferated reactively to TIF1-γ, could cause myositis in naïve B6 mice (the incidence: 50 %). Moreover, transfer of the purified CD8+ T cells, but not the purified CD4+ T cells, caused define myositis in naïve B6 mice (the incidences: 90 % and 0 %, respectively). 32microglobline-knockout mice, which lack major histocompatibility complex I to present antigens for CD8+ T cells, and perforin-knockout mice, in which CD8+ T cells lack cytotoxicity, developed significantly weaker myositis than wild-type mice (myositis scores: 0.13 ± 0.23, 0.30 ± 0.48, and 0.91 ± 0.70, respectively). In contrast, transfer of purified IgG from the TIF1-γ-immunized mice could not cause myositis in naïve B6 mice, moreover, tMT mice, which are B cell-deficient mice, developed myositis after TIF1-γ immunizations with no inferiority compared to wild-type mice.

Conclusions: TIF1-γ is an autoantigen with especial immunogenicity to induce experimental myositis. While the muscle injury is directly mediated by CD8+ T cells, but not CD4+ T cells and anti-TIF1-γ antibodies. Experimental myositis induced by immunizations of TIF1-γ should be a useful tool to investigate pathology of anti-TIF1-γ antibody -associated dermatomyositis.

### **Oral6 The temporal relationship between cancer and adult onset anti-transcriptional intermediary factor 1 antibody positive dermatomyositis**

#### A. Oldroyd

##### University of Manchester, Manchester, United Kingdom

Aim: This study aimed to characterise the 10 year relationship between anti-transcriptional intermediary factor 1 antibody (anti-TIF1-Ab) positivity and cancer onset in a large UK-based adult dermatomyositis (DM) cohort.

Methods: Data from adults with DM from the UKMYONET study were analysed. Anti-TIF-1-Ab positive and negative patients were included. Each patient was followed up until they either developed cancer or were censored due to death or end of follow up. UKMYONET recruitment began in 1999, and cancer occurrence linkage was carried out up until December 2016 through the UK Health and Social Care Information Centre. The cumulative incidence of cancer after DM onset was estimated according to Kaplan-Meier methods for the anti-TIF-1-Ab positive and negative cohorts. Hazard ratios for the time to cancer diagnosis by anti-TIF-1-Ab positivity were calculated using a Cox-regression model adjusted for age, gender and smoking status.

Results: Data from 263 DM cases were analysed, with a total of 3,252 person-years and a median 11 years follow-up; 55 (21%) of DM cases were anti-TIF1-Ab positive. Eighty percent of the anti-TIF1-Ab positive cohort were female, compared to 66% of the anti-TIF1-Ab negative cohort. Thirty one percent of both the anti-TIF1-Ab positive and negative cohorts smoked. After 10 years of follow up, a higher proportion of anti-TIF1-Ab positive cases developed cancer, compared to anti-TIF1-Ab negative cases: 38% vs 15% (hazard ratio adjusted for age, gender and smoking 3.4 [95% CI 2.2, 5.4]). All the detected malignancy cases in the anti-TIF1-Ab positive cohort occurred between 3 years prior to and 2.5 years after DM onset. No cancer cases were detected within the following 7.5 years in this group, whereas cancers were detected during this period in the anti-TIF1-Ab negative cases. Ovarian cancer was more common in the anti-TIF1-Ab positive vs negative cohort – 19% versus 2% respectively (p-value < 0.05). No anti-TIF1-Ab positive case aged under 39 years developed cancer, compared to 21 (53%) of those aged 39 years and over.

Conclusion: This study has helped to characterise the temporal relationship between anti-TIF-1-Ab positivity and cancer onset. Anti-TIF1-Ab positive-associated malignancy occurs exclusively within the three year period either side of DM onset, the risk being highest in those aged 39 years and over. Cancer types differ according to anti-TIF1-Ab status, and this may warrant specific cancer screening approaches.

### **Oral7 Pruritonegic mediators in skin samples of patients with dermatomyositis**

#### A. Vincze

##### University of Debrecen, Debrecen, Hungary

Objectives: Pruritus is a common symptom in systemic autoimmune diseases like dermatomyositis (DM). Recent researches have indicated that interleukin-31 (IL-31), IL-33, IL-6, or inflammatory cytokines, such as tumor necrosis factor (TNFα), peroxisome proliferator-activated receptor γ (PPARγ) and ion channels belonging to the transient receptor potential (TRP) family are involved in pruriception. We examined targeted gene expression analysis of lesional versus non-lesional skin samples of patients affected with active DM. We looked for correlations between the examined pruriceptive signaling molecules, disease activity and itching sensation of DM patients.

Methods: Gene expression of TNFα, PPARγ, IL-33, IL-6 and TRPV channels in lesional DM skin was evaluated by RT-qPCR and was compared with non-lesional DM skin samples. Pruritus and disease activity of DM was evaluated by the 5-d itch scale and Cutaneous Dermatomyositis Disease Area and Severity Index (CDASI), respectively. Statistical analysis was performed with IBM SPSS 20.0 software.

Results: Skin samples of 17 active DM patients were analyzed. We could show, that itching index in DM was positively correlated with CDASI score with a correlation coefficient of 0.82 (p < 0.001). TNFα gene expression was significantly higher in lesional DM skin than non-lesional DM skin (p=0.03), whereas PPARγ level was decreased, but this was statistically not significant. Normalized TNFα mRNA expression was positively (R= 0.605, p=0.022), while PPARγ negatively (R= -0.618, p=0.019) correlated with itch scale. Lesional IL-6 mRNA levels were associated with CDASI activity score (R=0.619, p=0.018). The mRNA levels of TRPV1-4 channels were not associated with 5-D itch score, but normalized TRPV1 and TRPV4 mRNA expressions were positively correlated with CDASI damage score (R=0.699, p=0.008; R=0.789, p=0.001). Interestingly, itching sensation of DM patients was not correlated with IL-33 mRNA levels measured in skin samples.

Conclusions: Our results argue for that TNFα and PPARγ might play a determining, but opposite role in DM-associated itch. Furthermore IL-6, TRPV1 and TRPV4 channels might participate in pathomechanism of cutaneous manifestation of the disease.

### **Oral8 High level of serum neopterin is associated with rapidly progressive interstitial lung disease and reduced survival indermatomyositis**

#### Q. Peng

##### China-Japan Friendship Hospital, Beijing, China

Objectives: Neopterin is primarily synthesized and released by activated macrophages/monocytes upon stimulation with interferon-γ and considered to be a marker of macrophage activation. The aim of this study was to analyze the serum levels of neopterin in patients with dermatomyositis (DM) in relation to clinical manifestations, laboratory data and patient prognosis.

Methods: One hundred and eighty-two consecutive dermatomyositis (DM) patients and 30 healthy controls were retrospectively enrolled in the study. Serum levels of neopterin were detected by using the ELISA method. Clinical and laboratory data, patient prognosis were obtained and analyzed in relation to serum neopterin.

Results: Serum levels of neopterin were significantly increased in DM patients (median 21.2 nmol/L, IQR 13.9-35.2 nmol/L) compared to healthy controls (median 4.3 nmol/L, IQR 2.9-5.6 nmol/L, P < 0.01). High serum neopterin levels were associated with anti-MDA5 antibody, rapidly progressive interstitial lung disease (RP-ILD), and cutaneous involvement including skin ulcer and heliotrope rash. Longitudinal assessment of serum samples revealed that serum neopterin levels were closely correlated with disease severity. In addition, a significant increase in serum neopterin concentration of non-survivors (median 38.7 nmol/L, IQR 23.5-65.3 nmol/L) was found comparing to that of survivors (median 19.0 nmol/L, IQR 12.5-29.0 nmol/L) (P < 0.01). ROC curves showed serum neopterin could distinguish non-survivors and survivors at an optimal cut-off level of 22.1 nmol/L with sensitivity and specificity of 0.804 and 0.625 respectively (P < 0.01). Kaplan-Meier survival curves revealed that DM patients with serum neopterin greater than 22.1 nmol/L had a significantly higher mortality compared to patient group with serum neopterin < 22.1 nmol/L (logrank P < 0.01). Multivariate Cox regression analysis identified high serum neopterin concentration to be an independent risk factor for poor prognosis in DM (adjusted HR=4.619, 95%CI: 2.092-10.195, P < 0.01).

Conclusions: Increased serum levels of neopterin were significantly associated with RP-ILD and reduced survival in DM patients, suggesting serum neopterin as a promising biomarker in the disease evaluation of DM. These findings highlight the role of cellular immune activation and macrophage activity in the pathogenesis of DM.

### **Oral9 Anti-MDA5 antibody-associated dermatomyositis: Three subgroups with different outcomes**

#### S. Toquet

##### Centre Hospitalier Universitaire de Reims, Reims, France

Objective: The antibody (Ab) anti-melanoma differentiation associated gene 5 (MDA5+) delineates a group of dermatomyositis (DM) patients with arthralgia and interstitial lung disease (ILD), sometimes rapidly progressive (RP-ILD) with life-threatening complications. The clinical symptoms of myositis can be absent. Characterizing different subsets of patients and their outcomes maybe helpful for the disease management.

Methods: We conducted a multicentric, retrospective study, and collected clinical, laboratory and imaging data of patients. MDA5+ patients presented a DM skin rash and/or arthralgia and/or ILD without other etiology in presence of anti-MDA5 Ab. Unsupervised analyses were performed and a classification and regression tree (CART) was used to determine items to classify patients.

Results: MDA5+ patients (n=121) were mainly female (67%), 49 years old, with in most of the cases a DM skin rash (87.5%), frequent ILD (77%; RP-ILD, 32.7%) and arthralgia (69%). A quarter (25.4%) of patients died. Within MDA5+ group, analysis showed three clusters. One was composed by patients with a RP-ILD (95%; p < 0.0001) and frequent mechanics’ hands (65%; p < 0.0001). One cluster corresponded to an “athro-cutaneo-form”, since in addition to the DM skin rash, arthralgia and/or arthritis were highly frequent (85%; p < 0.0001) whereas RP-ILD was unusual (9.5%; p < 0.0001). The last cluster was a group of patients with a “vasculo-cutaneomuscular-form” composed mainly by male (71.4%; p < 0.0001) with a severe skin vasculopathy (frequent Raynaud phenomenon, skin ulcers, digital necrosis and calcinosis) and clinical sign of myositis (weakness 71.3%; p < 0.0001). The mortality was very high (65%) in the ‘RP-ILD form’ contrasting with the very good outcome (no death) in the ‘athrocutaneo-form’ whereas the prognosis of ‘vasculo-dermatomyo-form’ was mild (19% of mortality). CART analysis was performed without including obvious signs of severity such as RP-ILD or mortality. The analysis showed that only three variables: Raynaud phenomenon, gender and articular symptoms enabled to predict in which cluster patients belong to (86% of good prediction).

Conclusion: MDA5+ patients present a systemic syndrome composed by three different forms with different outcomes, and only three variables permit to predict mortality.

### **Oral10 Risk stratification in patients with anti-melanoma differentiation-associated gene 5 antibody-associated interstitial lung disease treated initially with intensive combination regimen**

#### T. Gono

##### Nippon Medical School Graduate School of Medicine, Tokyo, Japan

Object: Anti-melanoma differentiation-associated gene 5 (MDA5) antibody-associated interstitial lung disease (anti-MDA5-ILD) is a unique dermatomyositis subset with a high prevalence of rapidly progressive ILD and poor outcomes. Patients in this subset are often treated with initial combination regimen consisting of high-dose corticosteroids, calcineurin inhibitor, and intravenous cyclophosphamide, but some patients have adverse outcomes despite intensive regimen. This study aims to identify predictors for mortality in patients with anti-MDA5-ILD treated with intensive regimens using a large multicenter cohort data. 

Methods: We selected 210 adult patients with anti-MDA5-ILD from a multicenter retrospective cohort of Japanese patients with Myositis-ILD (JAMI cohort). Risk factors for mortality were identified by multivariate analysis. We then created a risk stratification model using a combination of independent predictors.

Results: A total of 150 (71%) were treated with initial triple combination regimen, who demonstrated a poorer survival rate compared with those treated with less intensified regimen, suggesting that clinically severe cases were likely to be treated with more intensive regimen. In the triple combination regimen group, risk factors for mortality identified included SpO2/FiO2 ratio < 448 (odds ratio [OR] 6.6, 95% confidence interval [CI] 3.3-10.7) and age at diagnosis ≥ 63 years (OR 5.2, 95%CI 2.5-11.0). When the patients were stratified by the number of these independent risk factors, the mortality rates were 11%, 60% and 80% in patients with 0, 1, and 2 risk factors, respectively. Moreover, additional risk factors were identified within 64 patients without any risk factor, and included male (OR 4.1, 95%CI 1.1-18.3) and disease duration ≤ 1 month (OR 4.5, 95% CI 1.2-18.8). In this patient group, the mortality rates were 0%, 8% and 60% in patients with 0, 1, and 2 risk factors, respectively. These finding urged us to propose a risk stratification tree model in anti-MDA5-ILD patients.

Conclusions: This study successfully identified a patient subgroup of anti-MDA5-ILD who responds well to initial triple combination regimen. In other words, patients who are resistant to the intensified regimen and result in fatal outcome were identified and should require new therapeutic regimen and be candidates for future clinical trials.

### **Oral11 Quantitative high throughput screening of small molecule libraries to inhibit interferon beta-stimulated major histocompatibility complex class I in myositismuscle**

#### T. Kinder

##### National Institutes of Health (NIH), Bethesda, MD, United States

Objectives: The current immunosuppressive therapies for idiopathic inflammatory myopathies (myositis) often do not cure the condition and are accompanied by harsh side effects. We have recently initiated a program to find new or repurpose approved small molecule therapeutics for myositis. Although the cause of myositis is not known and the phenotype is heterogeneous, the abundance of type 1 interferon (IFN) and major histocompatibility complex class I (MHC class I) observed in myositis muscle have been hypothesized to contribute to pathology. We have developed a series of in vitro assays to perform quantitative high throughput screening (qHTS) to identify inhibitors of the IFN-stimulated expression of MHC class I in muscle with the goal of reducing inflammation and autoimmunity in myositis.

Methods: The primary screen involves immunofluorescence of MHC class I (HLA-ABC) in immortalized human myoblasts stimulated in 1536 well-plates with IFN-beta and treated with large and diverse chemical libraries containing approved, well-characterized, or novel compounds, and then analysis by laser scanning cytometry and high content imaging. Active molecules displaying concentration-response profiles for inhibition of HLA-ABC expression will be validated in primary myoblasts from myositis patients by both immunofluorescence and RT-qPCR of several HLA genes.

Results: We have developed both high throughput RT-qPCR and immunofluorescence assays for HLA-ABC in immortalized human myoblasts, and have screened a small, well annotated library of 1280 pharmacologically active compounds. The primary immunofluorescence screen had an adequate Z’ score of 0.3 ± 0.1, was highly reproducible, and identified nine compounds that decreased MHC class I expression with minimal toxicity over the nM and uM range. These actives need to be validated in follow-up assays, and we plan to screen additional libraries such as a collection of approved drugs and larger novel compound libraries.

Conclusion: These efforts will be the first application of qHTS technology with a chemical genomics approach to interrogating the IFN-MHC response in myositis muscle in an effort to bring better therapies to myositis patients.

### **Oral12 Study of Tofacinitib in refractory dermatomyositis**

#### J. Paik

##### Johns Hopkins University, Baltimore, MD, United States

Objective: Dermatomyositis can be difficult to treat and it is common to fail 2 or more steroid sparing agents or high dose steroids. This open label 12 week study was conducted to evaluate the efficacy and safety of tofacitinib, a JAK inhibitor, in active, treatment refractory DM.

Methods: Tofacitinib was given as 11mg XR daily to 10 subjects. Subjects were washed out of any steroid sparing agent and not allowed more than 20 mg of prednisone daily prior to study entry. The primary outcome was the proportion of subjects meeting the definition of improvement (DOI) at 12 weeks, defined by the International Myositis Assessment and Clinical Studies (IMACS) as improvement of > 20% in 3 of 6 core set measures (CSM) with no more than 2 worsening by > 25% [(which cannot include the manual muscle testing (MMT)]. Cutaneous Dermatomyositis Disease Area and Severity Index (CDASI), steroid-sparing effect of tofacitinib, safety, and tolerability were the secondary outcome measures.

Results: Mean age at enrollment was 45.6 +/- 10.6 years and 7 of 10 (70%) were female. All subjects failed 2 or more steroid sparing agents. All 10 subjects met the primary outcome DOI at 12 weeks, with 5 of 10 (50%) demonstrating moderate improvement and 5 of 10 (50%) having minimal improvement based on the Total Improvement Score (TIS) of the Myositis Response Criteria. The median TIS was 40 [IQR 32.5, 47.5] indicative of at least moderate improvement in all 10 subjects. The secondary outcome of the mean change in CDASI activity score from baseline to 12 weeks was statistically significant (28 + 15.4 (baseline) vs. 9.5 + 8.5 (12 weeks), p=0.0005). Chemokine data (CXCL-9/10) on 9 subjects also showed a trend toward improvement with treatment but did not show a statistically significant change from baseline (CXCL9, p=0.09; CXCL10, p=0.06). Four of 10 (40%) were on prednisone 20mg/daily at entry and 3 of 4 (75%) were able to completely taper off all steroids. Seven subjects were positive for anti-TIF-1 gamma and 5 of 7(71%) were moderate responders while 2 of 7 (29%) were minimal responders. There were no serious adverse events during the study.

Conclusion: This is the first clinical trial of tofacitinib, a JAK-inhibitor, in dermatomyositis. It demonstrated evidence of strong clinical efficacy as measured by a validated myositis response criteria. Future randomized controlled trials should be considered to assess efficacy of JAK-inhibitors in dermatomyositis.

### **Oral13 A one-year study investigating the effect of specialised and intensive ADL training in patients with idiopathic inflammatory myopathies-preliminary results**

#### M. Špiritović

##### Institute of Rheumatology Prague, Prague, Czech Republic

Background: Idiopathic inflammatory myopathies (IIM) form a heterogeneous group of mostly subacute acquired diseases of muscles accompanied by muscle weakness caused by inflammation and immune changes in the affected muscles, resulting in a decrease in quality of life. The aim of our study was to determine the influence of specialized and intensive ADL training on muscle strength, endurance and quality of life of patients with IIM.

Objectives and Methods: The study included a total of 50 IIM patients who fulfilled the Bohan and Peter 1975 criteria and had skeletal muscle involvement. 27 patients were recruited into the intervention group (IG) and 23 patients into the control group (CG). Both groups received an educational material for home exercise, but only the IG underwent a half-year intensive training with a subsequent half-year follow-up period. Patients were assessed by a physician and a physiotherapist blinded to intervention at months 0, 3, 6, and 12, and parameters evaluating quality of life, muscle strength and endurance were recorded. Patients also filled out PRO (patient reported outcomes) questionnaires and provided blood for routine laboratory analysis and bio-banking. Data analysis was performed between groups and within the group.

Results: Compared to the observed statistically significant deterioration in the CG over the intervention period of 0-6 months, we found a statistically significant improvement in the IG in objectively assessed strength and endurance of muscles as well as in subjectively assessed functional abilities and depression. During the follow-up period, there was a significant deterioration or stagnation of the achieved positive results in the IG. However, improved functional ability during the intervention period persisted in the IG in the follow-up period as well. Only numerical improvements in the IG during the intervention compared to numerical deterioration in CG, that did not reach statistical significance, were observed in some subjectively assessed domains of QoL (SF-36) and fatigue (FIS – in physical dimension).

Conclusions: Our specialized and intensive ADL training led to a significant improvement in the observed parameters that was clinically significant in a substantial proportion of patients, and prevention of the expected worsening of muscle weakness and quality of life.

Acknowledgement: Supported by AZV-16-33574A, SVV for FTVS UK 2019-260466, MHCR 023728.

### **Oral14 The use of metabolomics to develop novel biomarkers for juvenile dermatomyositis**

#### J. Dvergsten

##### Duke University Medical Center, Durham, NC, United States

Objectives: In juvenile dermatomyositis (JDM), mitochondrial dysfunction has been postulated to contribute to skeletal muscle injury. This derangement as well as the resulting cellular injury lead to variations in levels of intermediaries and products of metabolism. The metabolic state of the muscle has not been characterized in a cohort of patients with JDM. In this pilot study, we proposed to analyze newly diagnosed JDM subjects as well as those with disease flare off medication to identify aberrant metabolic signatures in blood samples as compared to healthy controls.

Methods: We studied seven newly diagnosed JDM patients (mean age, 9 years) and three flare (mean age, 14 years) patients off medication (greater than 8 months) as well as 10 control subjects who were age- and gender-matched. A targeted mass spectrometry-based platform was used to measure amino acids, acyl carnitines, ceramides, and sphingomyelins. Traditional chemistry-based assays measured plasma ketones, non-esterified fatty acids, glucose, and lactate. Statistical analyses were performed using logarithmically-transformed concentrations. Principal components analyses (PCA) was used to reduce the dimensionality of metabolite data prior to t-tests for group comparisons.

Results: PCA produced six factors explaining 74% of the dataset variance. As compared to the control subjects, patients with newly diagnosed JDM or a flare of their disease off medication, exhibited higher factors scores for Factor 2 (P less than 0.02) which contained long chain acylcarnitines (mean log-transformed values, control/JDM): C16OH/C14DC (.0046/.0093), C18:1OH/C16:1DC (.0053/.0108), C18OH/C16DC (.0077/.0131), C18:1DC (.008/.015), C20OH/C18DC (.0079/.0146). There were no group differences in scores for factors containing medium chain acylcarnitines (Factor 1), branched chain amino acid (BCAA) and BCAA catabolism by-products (Factor 3), amino acids (Factor 4), four carbon acyl carnitines with proline, arginine, and histidine (Factor 5), and several unsaturated acylcarnitines (Factor 6) all with P greater than 0.05.

Conclusions: In active JDM (new diagnosis and flare of disease), there were greater systemic concentrations of long chain acylcarnitines. These intermediates may reflect a disease-related block in the early steps of beta-oxidation and at least one component of the JDM-associated mitochondrial dysfunction. Future steps in this study include comparison of JDM muscle versus JDM blood metabolites from this cohort of newly diagnosed patients as well as JDM muscle versus control muscle metabolites to identify additional aberrant patterns of expression and endeavor to discover.

### **Oral15 Endothelial biomarker profiles reflect heterogeneity of juvenile dermatomyositisand may predict disease course**

#### J. Wienke

##### UMC Utrecht, Utrecht, Netherlands

Objectives: Vasculopathy is an important hallmark of juvenile dermatomyositis (JDM). We aimed to relate biomarker profiles of markers reflecting endothelial dysfunction and inflammation to clinical heterogeneity at disease onset and disease course.

Methods: 39 analytes reflecting endothelial dysfunction and inflammation were measured by multiplex immunoassay in 30 serum samples of JDM patients from Chicago, taken before start of treatment. Patients were clustered based on their biomarker profile. Disease activity, auto-antibodies, nailfold end row loops (ERL) and disease course (e.g. flare and time to remission) were related to biomarker profiles.

Results: Unsupervised hierarchical clustering of patients based on biomarker profiles revealed three distinct clusters: Cluster A showed the highest expression of inflammatory markers BLC, MIP-3beta, TNFR2, IL-18, galectin-1 and YKL-40 and interferon-related markers galectin-9, IP-10, MIG and MCP-1, next to endothelial activation marker sICAM. Patients in cluster B had intermediate levels of these markers, but significantly higher galectin-1, galectin-9, TNFR2, TWEAK, MDC, endoglin, sVEGFR1, C-TACK, PlGF, IL-18, OSF-2 and VEGF than cluster C. Principal component analysis revealed that muscle disease activity was the most important clinical factor corresponding to the different biomarker profiles: patients in clusters A had the most abnormal CMAS (p=0.01) and DAS muscle (p=0.05) compared to the other clusters. MCP-1, YKL-40, Angiopoietin-2, MIP3beta, BLC, OSF-2, sVCAM and sICAM had the highest correlations with muscle (related) symptoms. Skin DAS did not differ between the clusters and correlated only with thrombomodulin, a biomarker for thrombosis/microangiopathy (rs=0.42, p=0.02). Anti-NXP2+ patients had higher muscle DAS and MCP-1 than anti-TIF1g+ patients, but lower C-TACK than antibody-negative and anti-TIF1g+ patients (all p < 0.05). VEGF was higher in antibody-negative than anti-TIF1g+ patients (p=0.04). Cluster C had significantly higher ERL scores than clusters A&B, indicating milder vasculopathy. Lower ERL scores corresponded to higher levels of known vasculopathy-markers endoglin (rs=-0.67,p < 0.0001) and TSP-1 (rs=-0.41, p=0.03) and lower levels of sICAM (rs=0.46, p=0.01).

The frequency of flares in the first year did not differ between the clusters, but galectin-3 levels at onset were significantly higher in patients developing a flare later (p=0.02). Patients in cluster A took longer to reach remission off medication than clusters B&C and high levels of IL-18, BLC, MIP-3beta, galectin-9, TNFR2, MCP-1, IP-10 and MIG predicted a longer time until remission off medication.

Conclusion: JDM patients can present with a spectrum of muscle disease activity, which relates to biomarker expression profiles that may be useful to predict elements of the disease course.

### **Oral16 The relationship of pain, fatigue and emotional distress with quality of life in juvenile myositis**

#### K. Ardalan

##### Northwestern University, Evanston, IL, United States

Objectives: Juvenile myositis (JM) is an autoimmune disease that negatively impacts quality of life (QoL) outcomes via muscle weakness and vasculopathic rashes. The relative contribution of pain, fatigue, and emotional distress to QoL in JM is incompletely understood. In this cross-sectional study, we assessed the relationships of pain, fatigue and emotional distress to QoL in JM.

Methods: JM patient-parent dyads (5-17 yo) were enrolled at routine visits. Descriptive statistics were calculated for demographic and clinical variables. Generic QoL was measured by PedsQL Generic Core Scales (PedsQL-GC) self-report (8-17yo) and parent-proxy report (5-17yo), while Patient-Reported Outcomes Measurement Information System (PROMIS®) patient (8-17yo) and parent-proxy (5-17yo) fixed short forms were used to assess depressive symptoms, anxiety, fatigue, and pain interference. Since PedsQL-GC were not normally distributed, multivariable quantile regression was performed with PROMIS domains on the median PedsQL-GC measures.

Results: Seventy-five JM patient-parent dyads were enrolled, with typical demographic features (n = 71 [94.7%] with dermatomyositis, n = 59 [78.7%] female, n = 59 [78.7%] white, median age = 11.7 yo [IQR: 8.1-14.3]). Patient PROMIS Fatigue was significantly associated with PedsQL-GC Physical (regression coefficient -0.795, 95% confidence interval [CI] -1.146, -0.134, p = 0.021) and Psychological scores (regression coefficient -0.643, 95% CI -1.073, -0.258, p = 0.003) across most quartiles. Parent-proxy PROMIS Fatigue was significantly associated with PedsQL-GC Physical scores across all quartiles (regression coefficient -1.082, 95% CI -1.512, -0.763, p < 0.001), but this relationship was not as consistent for PedsQL-GC Psychological scores (p = 0.29). While patient/parent-proxy PROMIS Depressive Symptoms, Anxiety, and Pain Interference were significantly associated with most PedsQL-GC Physical and Psychological score quartiles in univariable models (not shown), these relationships did not persist in multivariable models.

Conclusion: Our findings demonstrate a uniquely strong relationship between fatigue and physical QoL as measured by both patients and parents. Patient and parent perceptions differed with regards to the relationship of fatigue to psychological QoL, reinforcing the need for both respondents to be engaged. The relationship between disease activity, treatments, fatigue, and QoL warrants further study, as fatigue may be an important target for interventions to improve QoL.

### **Oral17 Juvenile dermatomyositis – data of a national pediatric rheumatology database**

#### C. Sengler

##### German Rheumatism Research Center, Berlin, Germany

Objectives: Juvenile dermatomyositis (JDM) is the most common idiopathic inflammatory myopathy in children. We were interested how JDM presents clinically, how it is diagnosed and treated in Germany nowadays.

Methods: Children and adolescents with JDM who were documented by means of a disease-specific physician questionnaire and a patient questionnaire in the national pediatric rheumatology database (NPRD) in the years 2014 – 2016 were considered for this analysis. The method of diagnosis, clinical manifestations until documentation, patient-reported outcomes and current treatments were assessed.

Results: Data were available for 186 patients with JDM, 69% were female. Mean age at disease onset was 6.8 years (SD 3.5), mean time span between first symptoms and first contact to a pediatric rheumatologist was 6.1 months (SD 8.9), mean age at documentation was 11.5 years (SD 4.4). The JDM diagnosis was based on the following pathological/positive findings: Histology 44/147 (30%), electromyography 28/144 (19%), magnetic resonance imaging 103/148 (70%). Creatine kinase was elevated at time of diagnosis in 126 of 148 patients (85%). At documentation or after a mean disease duration of 5 years (SD 3.9), the physician’s global assessment of disease activity (PGA, NRS, range 0 – 10, best 0) was 1.6±2.3 (mean); the manual muscle test (MMT, range 0 – 80, best 80) was 69.7 (SD 19) and the mean disease activity score (DAS, range 0 – 20, best 0) was 4.4 (SD 4.7). Patients rated the following outcomes (NRS, 0 – 10) as follows: mean disease activity 2.3 (SD 2.8), pain 1.5 (SD 2.5), fatigue 1.6 (SD 2.7), overall well-being 2.0 (SD 2.4); the mean CHAQ was 0.5 (SD 0.8). At documentation, 30% (50/166) of patients received oral glucocorticoids < 0.2 mg/kg and 13% (21/166) oral glucocorticoids ≥ 0.2 mg/kg body weight. The following substances were mainly used as conventional synthetic disease modifying anti-rheumatic drugs (csDMARD): Methotrexate in 77/171 (45%), hydroxychloroquine in 49/171 (29%), mycophenolatemofetil in 4/171 (2%). Only 7 patients received biologics, 6 rituximab and 1 etanercept. Intravenous immunoglobulins had been administered to 33/163 patients (20%) in the last 12 months.

Conclusion: Demographic and clinical parameters of patients with juvenile dermatomyositis in Germany are comparable to JDM-cohorts in other countries. MRI has gained diagnostic importance and is used more than twice as often as biopsy. Mean disease activity after mean disease duration of 5 years is low but almost half of the patients received methotrexate and glucocorticoids at last consultation.

### **Oral18 Mitochondrial dysfunction: A novel treatment focus in juvenile dermatomyositis**

#### M. Wilkinson

##### University College London, London, United Kingdom

Objectives: There is a significant unmet need to develop more effective targeted treatments for juvenile dermatomyositis (JDM). By identifying differentially expressed (DE) genes and pathways using RNA-seq we can explore novel underlying pathogenic mechanisms of disease; this study aimed to identify dysregulated biological processes upstream of the type I interferon (IFN1) signature.

Methods: Peripheral blood samples were obtained from patients enrolled in the Juvenile Dermatomyositis Cohort and Biomarker Study (JDCBS) pre-treatment (n=13) and 11.8 [11.3-13.2] months on-treatment, (n=13) including n=10 matched samples, and age/sex-matched child healthy controls (CHC) [n=8]. CD4+, CD8+, CD14+ and CD19+ cells were sorted by flow cytometry from PBMC, RNA extracted and sequenced. Gene set enrichment analysis (GSEA) and gene ontology (GO) term enrichment analyses were performed. To investigate mitochondrial function, complex-I activity was measured and normalised to mitochondrial content measured by citrate synthase activity. Normalised complex-I activity was calculated comparing adult healthy control (AW) [n=8] and JDM [n=5] samples. Cell-free circulating DNA was isolated from plasma. Using standard curve qPCR the isolated DNA was amplified to quantify mitochondrial DNA (mtDNA, expressed as copy number of the mitochondrial gene, MT-CO3). mtNDA copy number per ml of blood was quantified for CHC [n=6] and JDM [n=55].

Results: RNA-seq confirmed a strong IFN1 signature (normalized enrichment scores 2.39, 2.84 , 2.88 and 3.41 (all p < 0.001) for CD4, CD8, CD19 and CD14 cells, respectively). Interestingly, CD14+ cells had the most differentially expressed (DE) genes among all cell types from on-treatment JDM compared to CHC (1,594 genes). GO analysis of these genes identified over-representation of GO-terms involved in mitochondrial function. Particularly genes involved in mitochondrial function were abnormally expressed in both pre- and on-treatment CD14+ compared to controls, suggestive of mitochondrial dysfunction (MtD) not corrected by current treatment. Reduced expression of mitochondrial-encoded genes was associated with increased expression of IFN1-stimulated genes in JDM CD14+ monocytes (R=-0.87, p=0.003). To confirm MtD we showed that complex-I activity was decreased in JDM monocytes compared to AW. As a marker of MtD, cell-free circulating mtDNA levels were increased in JDM plasma mtDNA compared to CHC (p=0.023).

Conclusion: The transcriptomic study in combination with biochemical and molecular measures of mitochondrial function have identified mitochondrial dysfunction as a novel pathogenic mechanism in JDM. These preliminary data need to be supported by validation and functional work to investigate whether there is a mechanistic relationship between interferonopathy and MtD, which may represent a potential therapeutic target.

### **Oral19 Differential peripheral IFN score expression in juvenile dermatomyositis vs. Mendelian interferonopathies**

#### H. Kim

##### National Institutes of Health (NIH), National Institute of Arthritis and Musculoskeletal and Skin Diseases (NIAMS), Bethesda, MD, United States

Objectives: JDM is a complex autoimmune disease with an interferon (IFN) signature. Myositis-specific autoantibodies (MSA) define phenotypic features of JDM. Proteasome mutations are associated with systemic inflammation, panniculitis, lipodystrophy, myositis and a strong IFN signature in CANDLE. Mutations in STimulator of IFN Genes (STING) cause an autoinflammatory disease (SAVI) with vasculopathy, systemic inflammation, lung disease, acral necrosis, and a notable IFN signature. Assessment of an IFN gene score (IFN_s) in JDM versus CANDLE and SAVI may help elucidate the role of IFN in JDM. Evaluation of IFN_s by MSAs and disease activity will assess possible differential role of IFN and potential biomarker performance.

Methods: A twenty-eight IFN-regulated gene score previously developed in CANDLE patients was assessed in prevalent JDM patients with active disease versus CANDLE, SAVI, NOMID (IL-1 mediated autoinflammatory control), and healthy controls (HCs) via whole blood RNA on Nanostring (Seattle, WA). IFN_s among disease groups and MSAs was evaluated by Mann-Whitney. Principal component (PC) analysis (PCA) was performed to assess IFN_s by diagnosis. IFN_s was correlated with physician global activity (PGA), organ system-specific disease activity, manual muscle testing (MMT28), and physician global damage (PGD) on a visual analog scale (VAS).

Results: IFN_s for JDM (n=58) was significantly higher than NOMID (n=18) and HC (n=26) and significantly lower than SAVI (n=7) and CANDLE (N=11) (p< 0.05). Among 4 MSA groups (anti-TIF1 (n=21), NXP2 (n=11), MDA5 (n=11), MSA-negative (n=9)), IFN_s was significantly higher than NOMID and HC. The IFN_s did not differ in anti-MDA5 and MSA-negative JDM patients from CANDLE or SAVI, while anti-TIF1 and NXP2 had lower IFN_s. PCA distinguished SAVI and CANDLE from HC and NOMID, with JDM overlapping more with SAVI than CANDLE in PC2 and PC3. Anti-MDA5 and MSA-negative groups had increased overlap with SAVI versus CANDLE by PCA in PC2 and PC3, more than anti-TIF1 and NXP2. In JDM, IFN_s significantly correlated with PGA, extramuscular VAS, and MMT28 (rs=0.33-0.47), without correlation to PGD. Additional trends were observed within specific MSA groups, including higher correlation with cutaneous VAS in anti-TIF1 (rs=0.62) and skeletal VAS in anti-NXP2 (rs=0.94) patients.

Conclusions: Though the peripheral blood IFN-regulated gene score in JDM is elevated and correlates with disease activity, it is lower than Mendelian interfernopathies (CANDLE, SAVI). PCA demonstrates greater overlap with SAVI than CANDLE. Assessments also show some variation by MSA group.

Funding: Cure JM Foundation, Intramural Research Program of NIH, NIEHS, NIAID, NIAMS, CC.

### **Oral20 Juvenile inflammatory myopathy with anti-MDA5 auto-antibodies: A specific subgroup demonstrating differentially enhanced upregulation of interferon-alpha signaling**

#### I. Melki

##### Robert Debré Hospital, Paris, France

Introduction: Juvenile dermatomyositis (JDM) and juvenile overlap myositis (JOM) represent heterogeneous subtypes of juvenile idiopathic inflammatory myopathy (JIIM). Chronic evolution may occur in 60% of cases, and morbidity/mortality is substantial.

Objectives: Clinical, biological and histological characterisation, particularly relating to type I interferon (IFN I), of JIIM with anti-MDA5 auto-antibodies at presentation (group 1) in comparison to other JIIM (group 2).

Methods: Retrospective and prospective study of patients with JIIM ascertained from three French paediatric rheumatology reference centres between 2013 and 2018. Severe forms were defined as those requiring ICU, and/or more than two treatment lines and/or with fatal outcome. IFN I pathway activity was assessed by dosage of interferon alpha (IFNa) using digital ELISA (Simoa) measured in patient sera and myoblast lysate, and mRNA expression of interferon stimulated genes (ISGs) assessed using RTqPCR in whole blood.

Results: 71 patients were included, 13 (group 1) and 58 (group 2). Group 1 patients demonstrated more arthritis (100% vs 24%), cutaneous ulcerations (42% vs 26%), interstitial lung disease (ILD) (33% vs 14%), and milder muscular involvement (median of CK: 96 vs 149 UI/l; MMT 79/80 vs 70/80 and C-MAS 50/52 vs 38/52). Total muscle biopsy score was lower in group 1 (5.5 vs 20/27). Severe forms were not statistically different between the groups; 1 patient died in group 1 (rapidly progressive ILD – RPILD) vs 3 patients (group 2: no RPILD). 133 IFN signatures (ISGs) and 216 IFNa Simoa measurements were recorded. ISGs and IFNa were higher in group 1 than 2 (median interferon score 21 vs 7.4, and median IFNa protein 3807.5 vs 49.6 fg/ml). IFNa levels in patient myoblasts were similar to controls in the single group 1 patient assessed, whilst those in group 2 were higher than controls (10 patients). Treatment with steroids, tacrolimus, rituximab and immunoadsorption in 1 group 1 patient followed serially over 19 months correlated with the disappearance of MDA5 auto-antibodies, the normalisation of IFNa levels and subsequent complete remission, sustained 7 and 5 months after stopping immunoadsorption and steroids respectively.

Conclusion: This study indicates a higher frequency of cutaneous ulcerations, arthritis and ILD and milder muscular involvement in JIIM with positive MDA5 auto-antibodies compared to other JIIM. Our data suggest an important role of systemic IFNα in the pathology of the disease, especially in the anti-MDA5 auto-antibody positive subgroup. In severe and refractory forms of JIIM, IFNα may represent a therapeutic target.

### **Oral21 Identification of six dermatomyositis subgroups using principal component analysis-based cluster analysis**

#### H. Zhu

##### Peking Union Medical College Hospital, Chinese Academy of Medical Sciences, Beijing, China

Objective: Dermatomyositis (DM) is a heterogeneous disease with a wide range of clinical manifestations. The aim of the present study was to identify the clinical subtypes of DM by applying cluster analysis.

Methods: We retrospectively reviewed the medical records of 720 DM patients and selected 21 variables for analysis, including clinical characteristics, laboratory findings, and comorbidities. Principal component analysis (PCA) was first conducted to transform the 21 variables into independent principal components. Patient classification was then performed using cluster analysis based on the PCA-transformed data. The relationships among the clinical variables were also assessed.

Results: We transformed the 21 clinical variables into 9 independent principal components by PCA and identified 6 distinct subgroups. Cluster A was composed of two sub-clusters of patients with classical or moderate DM. Cluster-B patients were older and had malignancies. Cluster C was characterized by interstitial lung disease (ILD), skin ulcer, and minimal muscle involvement. Cluster D included patients with prominent lung, muscle, and skin involvement. Cluster E contained DM patients with other connective tissue diseases. Cluster F included all patients with myocarditis and prominent myositis and ILD. We found significant differences in treatment across the six clusters, with clusters C, D, and E being more likely to receive aggressive immunosuppressive therapy.

Conclusion: We applied cluster analysis to a large group of DM patients and identified six clinical subgroups, underscoring the need for better phenotypic characterization to help develop individualized treatments and improve prognosis.

### **Oral22 Overexpression of type 1 IFN in skeletal muscle affects muscle function**

#### M. Morales

##### Binghamton University, Binghamton, NY, United States

Objectives: Many studies have confirmed a role for type 1 interferons (IFNs) in myositis pathogenesis. However, despite a type 1 interferon (IFN) “signature”—demonstrated by an upregulation of type 1 IFN-stimulated genes in human myositis patients, the effect of type 1 IFNs on muscle function is not fully known. Therefore, the objective of this study was to examine the effects of IFN-13 overexpression in normal and the MHC class 1 overexpressing mouse model of inflammatory myopathy.

Methods: To accomplish our objective, we used in-vivo electroporation of the tibalis anterior (TA) muscle to inject plasmids in 12-week old normal (H) or myositis (HT) female mice (n=4-8/group). Mice were anesthetized with ketamine and were injected with either an IFN-13 or GFP plasmid into the left TA and an empty/control vector into the right TA at an injection volume of 30 μl (1μg/ml concentration) by electroporation. Mice were euthanized 12 days post-electroporation and tissue was harvested for gene expression, QPCR, and force contraction analyses.

Results: Prior to examining the effect of IFN-13 in our mouse model, we confirmed a significant upregulation of IFN-stimulated genes in myositis (HT) mice compared to normal (H) mice. Both control and myositis mouse muscle showed type 1 IFN receptor expression by Western blotting. Type 1 IFN-signature genes (MXB and IFIT2) were increased in IFN-13 but not control plasmid-injected mice. We assessed specific force of the adjacent extensor digitorum longus (EDL) muscle because the TA muscle is damaged from electroporation. Myositis mice have significantly reduced EDL specific force compared to normal mice. Overexpression of IFN-13 in the TA of normal (H) mice significantly reduced muscle force in the adjacent (EDL) muscle compared to GFP- and empty vector-injected mice. On the other hand, overexpression modestly reduced specific force in HT mice injected with the IFN-13 plasmid.

Conclusions: The results from our study provide direct evidence for IFN-13 overexpression to produce muscle weakness in healthy mice. EDL force in healthy mice injected with IFN-13 was worsened to levels comparable to myositis mice, with no further exacerbation of muscle weakness in already diseased (HT) mice injected with IFN-13. Muscle weakness in HT mice was not worsened likely due to the already low levels of EDL max force observed in this muscle. Although we demonstrated muscle weakness and an increase in IFN-stimulated genes following IFN-13 overexpression, the underlying mechanism for these effects remain unknown and are currently being investigated.

### **Oral23 The role of low-density granulocytes (LDGs) and neutrophil extracellular traps (NETs) in the clinical profile of patients with idiopathic inflammatory myopathies**

#### J. Torres-Ruiz

##### National Institute of Medical Sciences and Nutrition Salvador Zubirán (INCMNSZ), Mexico City, Mexico

Objectives: The aim of this study was to evaluate the amount and function of LDGs and NETs in patients with IIM and correlate them with their clinical features.

Methods: We recruited 65 adult patients with dermatomyositis, polymyositis, and anti-synthetase syndrome according to their respective classification criteria. The percentage of LDGs was assessed by flow cytometry as those CD10+, CD15+ and CD14- cells in the mononuclear fraction. Also, we evaluated NETosis from LDGs and their LL-37 content by confocal microscopy after positive selection with magnetic beads. The serum NETs were quantified by ELISA, detecting the complexes of DNA with either neutrophil elastase (NE) or MPO. Complete clinical response was defined as the absence of muscular and extra muscular disease activity while taking immunosuppressive therapy.

Results: The most frequent diagnosis was adult onset dermatomyositis (DM) (67.6%). Patients with calcinosis (1.17 vs 0.32%, P=0.035) and dysphagia (1.66 vs 0.32%, P=0.028) had higher amount of LDGs. Likewise, LDGs correlated with disease activity scales (including MMT8 (R=-0.4, p=0.001, physician (R=0.319, P=0.011), and patient visual analogue scale (VAS) (R=0.3, P=0.013), ALT (R=0.31, P=0.011), AST (R=0.29, P=0.018), muscle damage VAS (R=0.33, P=0.001), cutaneous damage VAS (R=0.31, P=0.011), damage extension (R=0.32, P=0.009), damage severity (R=0.29, P=0.017). Only 23.9% of patients had clinical complete response and had significantly lower levels of LDGs (0.17 (0.08-0.49) vs 0.46 (0.23-1.35), P=0.030). Patients with DM in complete clinical response had lower levels of DNA-NE complexes (1 vs 1.13, P=0.037), whilst those with calcinosis had a higher amount (1.35 vs 1, P=0.04). There was a trend towards a higher amount of DNA-NE complexes in patients with capillary thrombosis (1.21 vs 1, P=0.06). The expression of LL37 in the LDG NETs from patients with DM in complete clinical response was higher in comparison to that of normal density granulocytes (NDG) (453 vs 35, P=0.0058) and in comparison with healthy donor NDG (453 vs 62, P=0.02).

Conclusions: LDGs are expanded in subjects with active IIM, vasculopathy (calcinosis) and dysphagia. LDGs correlate with disease activity and damage scales. Also, patients with active DM and those with calcinosis have a higher amount DNA-NE complexes. Furthermore, the expression of LL37 in NETs from LDGs is higher in patients with DM which could contribute to the type I IFN signature in this disease. Our findings support the role of NETs in the pathophysiology of IIM, especially in patients with DM and vasculopathy.

### **Oral24 Auto-antibodies targeting components of sarcolemma membrane repair: A pathogenic mechanism in inflammatory myopathies**

#### W. Jarjour

##### The Ohio State University, Columbus, OH, United States

Introduction: Idiopathic inflammatory myopathies (IIM) represent a group of disorders causing chronic inflammation and significant damage to skeletal muscle due to an unchecked autoimmune response. Two mouse models of IIM have recently provided new insights into the pathogenesis of the disease. The synaptotagmin VII null (Syt VII-/-) mouse is characterized by defects in membrane resealing and presents with a mild myositis at one month of age. A more robust model of IIM combines knock-out of Syt VII with a FoxP3 mutation resulting in a mouse with impaired membrane resealing and regulatory T-cell deficiency. Tripartite motif (TRIM) proteins are another family that has a critical role in membrane repair. Here, we explore the presence of a pathogenic mechanism involving sarcolemma membrane repair that is responsible for initiation and/or propagation of muscle inflammation.

Methods: Autoantibodies directed to TRIM family protein members were examined by immunoblotting and/or ELISA. Membrane repair was monitored ex vivo using an established assay where the membrane of individual muscle fibers of intact skeletal muscle bundles were injured using an infrared multi-photon laser. Confocal live cell imaging was used to record entry of FM4-64 dye, which only fluoresces once it enters the cell. To examine membrane fragility, mouse skeletal muscle was collected from wild type mice or RAG1-/- mice adoptively transferred with lymph node preparations from either Syt VII-/-/FoxP3-/Y mice or FoxP3-/Y. Tissue was analyzed by immunohistochemistry for evidence of inflammation and tissue injury.

Results: We have established by direct ELISA that auto-antibodies against TRIM72, a critical protein involved in sarcolemmal membrane repair, is elevated in IIM patient sera. We show, for the first time, that a deficiency in T-regulatory cells is not sufficient to induce sarcolemma fragility, however, purified antibodies against critical proteins that facilitate the sarcolemma repair process are sufficient to reduce membrane repair capacity. We also demonstrate that sarcolemma integrity is reduced in distal skeletal muscle in the absence of inflammation in our novel murine model of IIM. Moreover, we find that exogenous delivery of IIM (dermatomyositis, inclusion body myositis and polymyositis) patient serum containing antibodies to TRIM72 can compromise sarcolemma resealing in healthy skeletal muscle in an ex vivo laser injury assay.

Conclusion: These findings represent a novel mechanism in IIM whereby decreased sarcolemma integrity induces a vicious cycle of aberrant antigen presentation that directly contributes to the pathophysiology of idiopathic immune myopathies.

### **Oral25 Investigating chaperone-mediated selective autophagy in immune-mediated necrotizing myopathies**

#### N. Fischer

##### Charité - Universitätsmedizin Berlin, Berlin, Germany

Objective: Immune-mediated necrotizing myopathy (IMNM) is an idiopathic inflammatory myopathy that is histopathologically characterized by the simultaneous presence of necrotic myofibers, myophagocytosis and regenerating myofibers. Clinically, IMNM presents with subacute symmetric weakness of the proximal limbs. Non-rimmed vacuoles have been described in IMNM myofibers by others and us, but never been analysed in more detail. Vacuoles in other myopathies are associated with dysfunctional protein degradation systems of the cell. The objective of our study was to explore the nature and origin of the vacuoles and the involvement of protein degradation systems in IMNM.

Methods: Skeletal muscle biopsies of 54 patients with IMNM were analysed histopathologically. Additional qPCR studies on the transcription levels of genes involved in central protein degradation systems were conducted. sIBM patients and non-disease controls were used as comparison groups.

Results: Vacuoles were present in 77% of IMNM skeletal muscle biopsies. Vacuoles showed no staining for protein aggregates (amyloid, tau, TDP43, FUS, pa-Synuclein etc.), sarcolemmal proteins (Caveolin3, Dystroglycans, Dysferlin etc.) or lysosomal enzyme activity. Positive staining for autophagy-related proteins p62 and LC3 was detected in the great majority of IMNM muscles in a considerable amount of myofibres. Compared with healthy controls, significantly increased mRNA levels of p62 and LC3 were measured in IMNM muscle tissue, indicating active autophagy. Our investigations revealed a peculiar staining pattern of finely dotted, diffusely distributed p62 in the sarcoplasm of IMNM myofibers. This p62 staining pattern is present at a variable amount in 90% (n=45) of the biopsies analysed and stands in strong contrast to the focal p62 staining pattern found in sIBM. Investigating molecular chaperone systems involved in protein degradation, we found co-localization of p62-positive fibers with HSP70, alphaBCrystallin and Bcl-2-associated athanogene 3 (BAG3), strongly suggesting the specific activation of BAG3-mediated selective autophagy. P62 positive myofibers were decorated by sarcolemmal MHC class I, indicating the involvement of these very fibres into the immune response.

Conclusion: We show that non-rimmed vacuoles are frequently present in IMNM. We found that chaperone-associated autophagy is highly activated in IMNM and differs morphologically from the expression pattern of sIBM. Molecules of the BAG3-mediated selective autophagy pathway can be found in IMNM and co-localize with p62. Compared with sIBM, BAG3 mRNA levels are increased in IMNM, suggesting a specific role of selective autophagy in IMNM. We also show a direct link between primarily non-immune mechanisms like autophagy with the immune response in IMNM.

### **Oral26 Severe axial and pelvifemoral muscle damage in immune-mediated necrotizing myopathy evaluated by whole-body MRI**

#### O. Landon-Cardinal

##### Centre Hospitalier de l'Université de Montréal, Montréal, Canada

Objectives: Immune-mediated necrotizing myopathy (IMNM) is associated with early thigh muscle damage on MRI. Pattern and severity of muscle damage in other muscle groups and its relationship with clinical and serological features remain to be clarified.

Methods: IMNM patients with a whole-body MRI (WB-MRI) (n=42) were included as well as 60 IBM patients as controls. Each muscle groups (n=55) were evaluated and fat replacement was estimated in T1 sequence using the Mercuri score (1= normal; 2= mild, fat replacement < 30%, 3=moderate, 30-60%; 4=severe, > 60%). Overall lesion load was defined as the sum of all abnormal Mercuri scores (percentage) and lesion load quotient was defined as the overall lesion load divided by disease duration (years). Multidimensional analysis was performed to define homogenous groups of patients and to assess linear relationships between variables.

Results: IMNM patients (anti-HMGCR, n=25; anti-SRP, n=12 and seronegative, n=5) at WB-MRI were aged 48.1±15.8 years and had a disease duration of 9.8 ±8.1 years. Most severely affected muscle groups were located in the pelvifemoral (gluteus minimus: 2.71±1.15, gluteus medius: 2.6±1.17, great adductors: 2.55±1.27 and perineal: 2.43±1.42) and lumbar (lumbar extensors: 2.79±1.12) region. Unsupervised analysis showed two subgroups of patients: one with a mild lesion load (15±10%, n=32/42) and another with a severe lesion load (60±10%, n=10/42: p < 0.001). In the first group, the mean disease duration before WB-MRI was 6.8 ±6.0 years compare to 19.5±5.7 years in the second (p < 0.0001). Correlational studies demonstrated that disease duration was the most important predictor of muscle damage (lumbar r=0.76, and pelvifemoral muscle groups r ranging from 0.55-0.73; p < 0.01). Nonetheless, multivariate analyses – adjusted for disease duration, age at first symptoms and treatment initiation delay - demonstrated a more severe involvement of the great adductor (p=0.02) and the vastus lateralis (p=0.02) in female patients. No difference was found between overall lesion load quotient in IMNM compared to IBM (14±14.9 vs 9.4±8, p=0.3), but muscle involvement pattern was different. Muscle damage progression over time was more severe in the trapezius (p=0.05), infraspinatus (p=0.03), psoas (p < 0.001), iliac (p < 0.001), longus adductor (p=0.01) and pectinus (p < 0.001) in IMNM.

Conclusion: IMNM is associated with a severe axial and pelvifemoral muscle damage. Disease duration and gender status are important predictors of muscle damage. IMNM and IBM patients have a comparable overall lesion load, but progression was more severe in some muscle groups.

### **Oral27 Role of MircoRNAs in immune-mediated necrotizing myopathy through regulation of myogenic differentiation-associated genes**

#### Y. Zuo

##### China-Japan Friendship Hospital, Beijing, China

Objectives: Immune-mediated necrotizing myopathy (IMNM), as one of the subtypes of idiopathic inflammatory myopathy (IIM), is characterized by relatively severe proximal weakness, myofiber necrosis with minimal inflammatory cell infiltrate on muscle biopsy. MircoRNAs (miRNAs) regulate a wide range of developmental and physiological cellular processes including differentiation, proliferation, growth and apoptosis. In the past ten years, identification of differentially expressed miRNAs in muscle biopsy samples (myomiRs) from patients with inflammatory myopathies caused that miRNAs to be considered as new potential molecular pathogenesis. The aim of this study was to examine IMNM-related miRNAs and their target genes, as well as potential mechanisms involved in the onset of IMNM.

Methods: Multiple myomiRs which have been reported abnormally expressed in skeletal muscle or myocardial tissue were screened and qRT-PCR was conducted in muscle biopsy samples of 13 IMNM patients and 6 healthy controls to validate differentially expressed myomiRs in IMNM. Bio-informatic analysis was used to predict target genes of miRNAs and double-luciferase reporter assay to confirm. Several tests including qRT-PCR, immunofluorescence, western blot analysis were performed in vitro and in vivo to further explore possible pathogenesis of IMNM.

Results: The levels of miR-23a-3p, miR-146a-5p and miR-150-5p were significantly down-regulated in IMNM patients compared with healthy controls, and the predicted target gene of the three differentially expressed miRNAs was RNA binding protein, HuR, which specific interacting with and regulating the stability of mRNA of myogenic regulatory factors (MRF) including MyoD and Myogenin. HuR mRNA expression was significantly decreased in muscle tissue of IMNM patients compared with healthy controls (p=0.016). Neural cell adhesion molecule (NCAM) was used as the marker of regenerating muscle fibers. Immunofluorescence histochemical double staining technique was performed with specific antibodies against HUR and NCAM in muscle biopsy samples of IMNM patients. Staining results showed that HUR expressed in the nucleus of mature myocytes and in the cytoplasm of regenerating muscle cells. Furthermore, human myoblasts were cultured and then induced to differentiate. At the onset of myogenesis in differentiating human myoblasts, immunofluorescence showed HuR cytoplasmic abundance increased dramatically, returning to a predominantly nuclear presence upon completion of myogenesis, and staining results were consistent with the observation by western blot analysis.

Conclusion: MiR-23a-3p, miR-146a-5p and miR-150-5p can influence human myoblast differentiation by regulating the expression of HUR and possibly participate in the pathogenesis of IMNM.

### **Oral28 The interferon gene signature in dermatomyositis, the anti-synthetase syndrome, immune-mediated necrotizing myopathy, and inclusion body myositis: A comparative study**

#### I. Pinal-Fernandez

##### National Institutes of Health (NIH), National Institute of Arthritis and Musculoskeletal and Skin Diseases (NIAMS), Bethesda, MD, United States

Objectives: Activation of the type I interferon (IFN1) pathway is a prominent feature of dermatomyositis (DM) muscle and may play a role in the pathogenesis of this disease. However, the relevance of the IFN1 pathway in patients with other types of myositis, such as the antisynthetase syndrome (ASyS), immune-mediated necrotizing myopathy (IMNM), and inclusion body myositis (IBM), is largely unknown. Moreover, the activation of the type II interferon (IFN2) pathway has not been previously explored in myositis. In this study, our objective was to analyze both IFN1 and IFN2 pathway activation in muscle biopsies obtained from patients with different forms of myositis.

Methods: RNAseq was performed on muscle biopsy samples from 119 patients with DM, IMNM, ASyS, or IBM as well as on 20 normal muscle biopsies. The expression of IFN1- and IFN2-inducible genes was compared between the different groups.

Results: The expression of IFN1-inducible genes was high in DM, moderate in ASyS, and low in IMNM and IBM. In contrast, the expression of IFN2-inducible genes was high in DM, IBM, and ASyS but low in IMNM. The expression of IFN-inducible genes was correlated with indicators of disease activity. Of note, ISG15 expression levels alone performed as well as composite scores relying on multiple genes to monitor activation of the IFN1 pathway in myositis muscle biopsies.

Conclusions: IFN1 and IFN2 pathways are differentially activated in different forms of myositis. This observation may have therapeutic implications since immunosuppressive medications may preferentially target each of these pathways.

### **Oral29 Evaluation of a novel particle-based assay for detection of myositis specific antibodies**

#### K. Malyavantham

##### Inova Diagnostics, San Diego, CA, United States

Objectives: Myositis specific antibodies (MSA) represent important diagnostic tools and also help stratify idiopathic inflammatory myositis (IIM) patients with particular clinical features, treatment responses, and disease outcomes. Standardization of MSA detection is of high importance because these antibodies also have the potential to be used in classification criteria. The objective of this study was to evaluate the clinical performance of a novel particle based multi-analyte technology (PMAT) for the detection of MSA.

Methods: The study included 464 patient samples collected at Hospital Vall d’Hebron, Univeristy of Barcelona, most of whom had a diagnosis of IIM (n=264). As controls, samples from patients with myositis like conditions (ML, n=20), rheumatoid arthritis (RA, n=33), systemic lupus erythematosus (SLE, n=40), Sjögren’s syndrome (SjS, n=25), infectious diseases (ID, n=40) and healthy individuals (HI, n=42) were included. All samples were tested using a novel fully automated particle-based multi-analyte technology (PMAT, Inova Diagnostics, research use only; Jo-1, Mi-2b, TIF1y, PL-12, SAE, EJ, MDA5, HMGCR, PL-7, SRP, NXP2) which utilizes paramagnetic particles with unique signatures and a digital interpretation system.

Results: The sensitivity/specificity of the individual MSA were: 19.7%/100% (Jo-1), 7.2%/100.0% (Mi-2), 3.0%/99.0% (NXP2), 3.8%/100.0% (SAE), 2.7%/100.0% (PL-7), 1.9%/99.5 (PL-12), 1.1%/100.0% (EJ), 15.5%/99.5% (TIF1y), 8.3%/98.5% (MDA5), 6.1%/99.0% (HMGCR) and 1.9%/98.5% (SRP). Of all IIM patients 136/262 tested positive for at least one of the MSA. In the individual control groups, 0/20 (0.0%) of ML, 2/33 (6.1%) of RA, 5/40 (12.5%) of SLE, 2/25 (8.0%) of SjS, 2/40 (5.0%) of ID and 1/42 (2.4%) of HI were positive for at least one MSA. Most of the control samples that tested positive had levels close to the cut-off (except one SRP and one PL-12). Only 6/264 (2.3%) IIM patients were positive for more than one antibody (MDA5/HMGCR, EJ/PL-7, 2 x MDA5/TIF1y, EJ/SAE, SAE/TIF1y). The overall diagnostic performance was: Sensitivity 68.2% (95% Confidence interval 62.3-73.5%), specificity 94.0% 95% CI 89.8-96.5) and odds ratio 33.8.

Conclusion: The novel PMAT used to detect a spectrum of MSA in IIM on a fully automated system showed good sensitivity and specificity in line with the known associations of MSA. Sensitivities and specificities of the individual MSA are within expected ranges.

### **Oral30 Optimization of muscle NMR imaged segmentation to best discriminate disease progression in adult patients with inflammatory myopathies**

#### H. Reyngoudt

##### Institute of Myology, Paris, France

Objectives: Fat fraction (FF), as calculated from water-fat (Dixon) NMR images, is a largely accepted muscle imaging biomarker, which has been proposed as an outcome measure in most neuromuscular disorders these last few years. The question remains, however, as to whether specific muscle or muscle groups should be taken into consideration for longitudinal evaluation. Here, we compared three different neuromuscular disorders: immune-mediated necrotizing myopathy (IMNM), (sporadic) inclusion body myositis (IBM) and GNE myopathy (GNEM). The aim of this work was to compare whole-segment FF with individual muscle and muscle group FF values and identify the most efficient procedure to quantify disease progression, by comparing the standardized response means (SRM).

Methods: Sixteen IMNM patients (18-74 years), ten IBM patients (18-75 years) and 10 GNEM patients (26-74 years) were scanned twice within a one-year interval on a clinical 3T Siemens PrismaFit NMR system. A fat/water separation 3-point Dixon NMR sequence was acquired in thigh and leg. Regions of interest (ROIs) were drawn in the different individual muscles, in muscle groups, in weighted combinations of individual muscles and muscle groups, and the whole leg and thigh. Changes in FF (ΔFF) using all methods were compared using ANOVA. SRMs over 0.8 were evaluated as being highly responsive to ΔFF.

Results: In IBM, SRM values were > 0.8 for almost all approaches, which was less the case for GNEM and IMNM. Global FF of the thigh is very sensitive in IBM patients (SRM=1.3) although quadriceps FF gives slightly higher values (SRM=1.6). For GNEM patients, vastus lateralis (SRM=1.1) or quadriceps (SRM=0.9) are more sensitive to change in FF than global FF (SRM=0.7). In IMNM, global thigh FF gives also an SRM of 0.7 but semimembranosus FF seems a better candidate for evaluating the disease progression (SRM=1.5). Global leg FF is sensitive enough in both IBM and GNEM (SRM=0.8), but for IMNM gastrocnemius medialis (SRM=0.8) is the most sensitive candidate.

Conclusion: We demonstrated that the optimal muscle groups in terms of FF SRMs are different in three adult muscle disease. This suggests that individual muscle segmentation – a tedious step in using qNMR imaging to characterize muscle structural changes, is not always mandatory depending on the pathology. There has also been a recurrent quest by investigators for the most appropriate muscle (group) on which to focus the analysis. Still, as based on our results, generalization of the concept is premature in the present stage.

### **Oral31 Identification of novel auto-antibodies for idiopathic inflammatory myopathies using human proteome microarray**

#### L. Li

##### Peking Union Medical College Hospital, Chinese Academy of Medical Sciences, Beijing, China

Objectives: Idiopathic inflammatory myopathies (IIMs) are a group of clinically heterogeneous, inflammatory muscle disorders characterized by proximal and symmetric muscle weakness and multisystem involvement. Autoantibodies are important biomarkers for IIMs, aiding in diagnosis, classifying patients into more homogeneous groups, and understanding additional clinical complications and responses to treatment. We aimed to employed human proteome microarrays, each composed of about 20,000 unique human proteins, to identify IIMs-specific autoantibodies.

Methods: A Three-Phase strategy was used. To screen candidate autoantigens, in Phase I, 90 serum samples collected from 40 IIMs patients, 30 autoimmune disease controls and 20 healthy subjects were probed individually to human proteome microarrays. To verify these candidates, in Phase II, a focused array with candidate IIMs-associated autoantigens was constructed, and this was used to profile a much larger cohort, comprised of serum samples collected from 397 IIMs patients (100 polymyositis, 217 dermatomyositis, 45 cancer-associated myositis, and 35 juvenile dermatomyositis), 197 disease controls (40 systemic sclerosis, 39 systemic lupus erythematosus, 40 primary Sjogren's syndrome, 39 rheumatoid arthritis, and 39 other chronic diseases), and 98 healthy controls. In Phase III, sera with high signal values (value > 3 standard deviation of the mean of the healthy group) for verified proteins were validated using western blot analysis.

Results: Ninety-one candidate autoantigens that were significantly associated with IIMs were identified in Phase I. After verification in Phase II, glycine n-methyltransferase (GNMT) was considered as a new IIMs-specific autoantigen. Twenty-three polymyositis, ten dermatomyositis, six cancer-associated myositis, two juvenile dermatomyositis, and three disease controls had high signal values for GNMT, while no healthy subject showed high signal values. In Phase III, 14 of the 23 polymyositis, six of the ten dermatomyositis, four of the six cancer-associated myositis, one of the two juvenile dermatomyositis, and one of the three disease controls were positive for anti-GNMT autoantibodies using western blot.

Conclusion: Anti-GNMT autoantibodies serves as a novel biomarker for IIMs and is expected to help clinical diagnosis. The prevalence of anti-GNMT autoantibodies in different ethnic populations and the association between anti-GNMT autoantibodies and clinical features should be further studied.

### **Oral32 IFN level assessed by ultrasensitive detection technology in myositis patients: A promising biomarker of disease activity in dermatomyositis and anti-synthetase syndrome**

#### L. Bolko

##### Pitié-Salpêtrière University Hospital, Paris, France

Inflammatory idiopathic myopathies (IIM) is a heterogeneous group of disorders ranging from muscle specific auto-immune diseases to systemic ones (dermatomyositis (DM), anti-synthetase syndrome (ASyS), immune-mediated necrotizing myopathy (IMNM) and inclusion body myositis (IBM)). Recent insight into DM pathogenesis highlighted the role of type I interferon (IFN) and the level of IFN-pathway activity is linked to those of the disease. The aim of this study was to measure IFN seric level in the different groups of myositis using an ultrasensitive detection technology to evaluate IFNs as disease activity biomarker.

Methods: IIM patients were enrolled in a monocentric prospective cohort. Clinical and biological data were prospectively collected as well as sera and peripheral blood mononuclear cells. Disease activity was assessed by calculating the Physician Global Activity (PGA) for each patient. To measure IFNs levels, sera were analyzed by single molecule array technology (SIMOA). The expression of IFN-stimulated genes (ISG) was detected by quantitative RT-PCR assays, and IFN scores were asses by the median gene expression of the 5(ISG).

Results: One hundred and fifty three patients (52 DM, 43 ASyS, 34 IMNM and 24 IBM) and 34 age-matched healthy controls were included. IFN-a levels were higher in DM (0.06 [0.03-0.23] pg/ml, p < 0.05) and ASyS groups (0.06 [0.02-0.16] pg/ml, p < 0.05) compare to controls (0.02 [0.01-0.04] pg/ml). IFN-a levels were similar in IMNM (0.03 [0.01-0.08] pg/ml), IBM (0.02 [0.02-0.03] pg/ml) and controls groups. Concerning IFN-β, only DM subgroup have a higher median (6.7 [1.24-59.4] compares with controls (1.24 [1.24-1.24] p < 0.001). For IFN-γ, all the subgroups have a higher level of this cytokine (DM= 0.85 [0.5-1.9]). IFN-a levels were correlated to disease activity in DM (r=0.72, p < 0.0001) and ASS groups (r=0.60, p < 0.001). The accuracy of IFN-a level to discriminate active and non-active disease was excellent attested by an area of 0.82 (p < 0,001) under the ROC-curve (AUC). For an IFN-a level above 0.15 pg/ml, the sensitivity was 64% and specificity was 96%. In ASS group, the accuracy of the test was also good (AUC=0.89) but the sensitivity and specificity were lower (57% and 95% respectively). In naïve DM and ASS patients (n=12), IFN scores, considered as as the gold standard to measure IFN activation, were assessed. The correlation between IFN score and IFN-a levels was very good (r=0.76, p=0.005).

Concluson: IFNa is strongly correlated with disease activity in DM and ASyS.

### **Oral33 Analysis of muscle transcriptomic features in idiopathic inflammatorymyopathies**

#### H. Chen

##### China Japan Friendship Hospital, Beijing, China

Background: Idiopathic inflammatory myopathies (IIM) are a group of complicated heterogenous autoimmune diseases, to date, little is known about its skeletal muscle transcriptomic features.

Objective: Here, we performed muscle RNA-sequencing (RNA-seq) to discover the global muscle transcriptional signature of IIM based on myositis-specific antibodies (MSA) profiles and investigate the potential molecular pathway of IIM.

Methods: Muscle specimen were taken from 60 patients with IIM, 6 patients with non-IIM myopathies and 9 healthy controls. The serum samples were also obtained from these patients at the time of doing muscle biopsy. For RNA-seq, IIM was dissected into eight groups based on their MSA profiles: MSA and ANA negative (n = 4), anti-synthetase antibodies positive (n= 13), -MDA5 positive (n = 6), -NXP-2 positive (n = 7), -TIF-1γ positive (n = 10), -SRP or anti-HMGCR positive (n = 6), -Mi-2 positive (n = 7), MSA negative but anti-Ro-52 positive (n = 7). RNA from muscle specimen were extracted according to manufactory guide and sequenced using Illumina HiSeq2500. Quantitative real-time reverse transcription-polymerase chain reaction (qRT-PCR) was performed on RNA-sequencing samples and expanded samples to validate the results of RNA-seq.

Results: To define the global muscle signature of IIM, all IIM samples were compared to NC and total of 1637 transcripts were differentially expressed (log2 Fold Change > 1, Padj < 0.05). Unsupervised hierarchical clustering of these differentially expressed transcripts (DETs) revealed a prevalent interferon (IFN) signature and showed that 68 interferon-stimulated genes (ISGs) were significantly up-regulated in IIM. Then these 68 ISGs were used to cluster different MSA subgroups and distinct ISG expression was found. The mRNA expression levels of several ISGs (IFIH1, LAMP3, CMPK2, HERC6, IL4I1) in sequencing samples and expanded samples also confirmed the diverse ISG expression between different MSA subgroups. An IFN signature scoring system was established to quantify the IFN activity. Subsequently IIM can be classified into IFN-Dominant, IFN-Moderate and IFN-Weak respectively based on the IFN intensity and different MSA subgroup.

Conclusion: We revealed a prominent IFN signature and MSA-based ISG expression heterogeneity in IIM through muscle transcriptomics. Preliminary results showed that the IFN muscle signature may play a predominant role in some subgroups but not all IIM groups in the pathogenesis of IIM.

